# Image-based robot navigation with task achievability

**DOI:** 10.3389/frobt.2023.944375

**Published:** 2023-05-31

**Authors:** Yu Ishihara, Masaki Takahashi

**Affiliations:** ^1^ Graduate School of Science and Technology, Keio University, Yokohama, Japan; ^2^ Department of System Design Engineering, Keio University, Yokohama, Japan

**Keywords:** image-based navigation, mobile robot, path planning, optimal control, deep learning

## Abstract

Image-based robot action planning is becoming an active area of research owing to recent advances in deep learning. To evaluate and execute robot actions, recently proposed approaches require the estimation of the optimal cost-minimizing path, such as the shortest distance or time, between two states. To estimate the cost, parametric models consisting of deep neural networks are widely used. However, such parametric models require large amounts of correctly labeled data to accurately estimate the cost. In real robotic tasks, collecting such data is not always feasible, and the robot itself may require collecting it. In this study, we empirically show that when a model is trained with data autonomously collected by a robot, the estimation of such parametric models could be inaccurate to perform a task. Specifically, the higher the maximum predicted distance, the more inaccurate the estimation, and the robot fails navigating in the environment. To overcome this issue, we propose an alternative metric, “task achievability” (TA), which is defined as the probability that a robot will reach a goal state within a specified number of timesteps. Compared to the training of optimal cost estimator, TA can use both optimal and non-optimal trajectories in the training dataset to train, which leads to a stable estimation. We demonstrate the effectiveness of TA through robot navigation experiments in an environment resembling a real living room. We show that TA-based navigation succeeds in navigating a robot to different target positions, even when conventional cost estimator-based navigation fails.

## 1 Introduction

There is a strong demand for technologies that can easily manage and control multiple robotic agents. The cybernetic avatar concept proposed by a working group of the Japanese government’s cabinet office aims to develop such technologies and infrastructure by 2050 to engage the aging population (Cabinet Office Government of Japan, 2019). Images would allow human operators to efficiently control such robotic agents. Furthermore, certain tasks are easy to execute visually (e.g., navigating to a place where an image was taken). Therefore, in this research, we focus on an image-based action selection method for controlling robots.

Several image-based action selection methods have been proposed owing to recent advances in deep learning (Zhu et al., 2017; Codevilla et al., 2018; Kahn et al., 2018; Kumar et al., 2018; Pathak et al., 2018; Hirose et al., 2019b; Eysenbach et al., 2019; Chaplot et al., 2020; Shah et al., 2020; Terasawa et al., 2020; Chebotar et al., 2021; Ishihara and Takahashi, 2021). These methods select the action according to the image corresponding to the initial state *s*_start_ and/or the image corresponding to the goal state *s*_goal_. To select an action, a deep neural network-based controller trained end-to-end is commonly used (Zhu et al., 2017; Codevilla et al., 2018; Kahn et al., 2018; Kumar et al., 2018; Pathak et al., 2018; Hirose et al., 2019b). In a real task involving a robot, there are safety requirements, such as avoiding the entrances to undesired areas and avoiding collisions with obstacles. However, satisfying these requirements using a controller trained end-to-end is difficult because it requires the analysis of a trained controller. For the practical use of robots, it is preferable to avoid the need for additional analysis. Conversely, conventional path-planning algorithms can easily satisfy these requirements by planning motions (e.g., moving in a certain direction at a certain speed) using an environmental map. Recently, several path-planning algorithms that use images as input have been proposed (Eysenbach et al., 2019; Terasawa et al., 2020; Ishihara and Takahashi, 2021). Therefore, in this research, we also consider an image-based path planner as a robot controller.

In previous research studies on image-based path planning, a robot’s actions were selected by estimating the optimal cost-minimizing path to reach the goal, such as the shortest distance or time, using the *s*_start_ and *s*_goal_ images (Kahn et al., 2018; Eysenbach et al., 2019; Terasawa et al., 2020; Ishihara and Takahashi, 2021). To estimate the cost of a given path from input images, deep neural network models are trained using the trajectory data obtained from the environment. As long as the evaluation is accurate, a robot can select the appropriate actions and complete the task. However, such models require a large amount of correctly labeled data to accurately estimate the cost. In real robotic tasks, collecting such data is not always feasible, and the robot itself may require collecting it. In this study, we empirically show that when a model is trained with data autonomously collected by a robot, the estimation of such parametric models could be inaccurate to perform a task. Specifically, the estimation accuracy decreases in proportion to the maximum predicted distance to the goal. Hence, the higher the maximum predicted distance, the more inaccurate the estimation, and the robot fails navigating in the environment.

**FIGURE 1:**
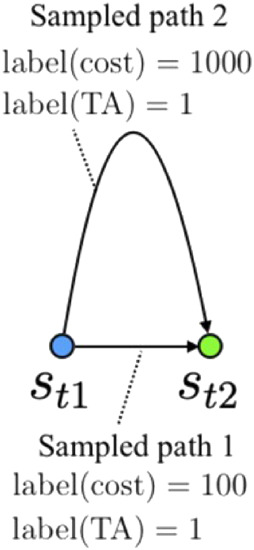
Example of labeled data generated from a random sampling of two states. If there exist two different sets of trajectory data that reach a goal state (*s*_*t*2_) from an initial state (*s*_*t*1_), the training data may contain incorrectly labeled (i.e., non-optimal) data (e.g., sample path 2) when training an optimal cost estimator. In contrast, when training a TA estimator, both data are labeled as 1.

To overcome this issue, we propose an alternative evaluation metric to evaluate paths planned for robots. We call this evaluation metric “task achievability” (TA), which is defined as *p*_*π*_(*s*_*T*_ = *s*_goal_|*s*_start_, *τ*): the probability that the robot will reach the goal state *s*_goal_ within time *T* with policy *π*, starting from an initial state *s*_start_ and following path *τ*. Even though this metric does not guarantee the optimality of the path to be navigated, we demonstrate that the path generated by TA is identical to the optimal cost-minimizing path. The advantage of using TA is shown in Figure 1. To train an optimal cost estimator, the dataset must consist only of optimal cost trajectories. However, it is difficult to create such a dataset by checking each image in the trajectory and removing non-optimal trajectories. Hence, training an optimal cost estimator is significantly affected by incorrectly labeled data, which leads to inaccurate cost estimations. In contrast, a TA estimator can use all the training data in the dataset without considering whether the trajectory in a dataset is optimal. Therefore, we expect the TA estimator to be stable and easy to train. In addition, because the TA metric’s value is always between 0 and 1 (i.e., the value is not proportional to the distance between states), we expect the trained model to estimate the value robustly.

In this research, we propose two types of estimation models, direct and indirect, to estimate the TA. The direct model trains a vision transformer (Dosovitskiy et al., 2021)-based model to output the probability *p*_*π*_(*s*_*T*_ = *s*_goal_|*s*_start_, *τ*) for each action in *τ*. The indirect model assumes deterministic dynamics and trains a state (image) prediction model *f*_pred_(*τ*, *s*_start_) that outputs the future state *s*_future_ and estimates the probability *p*_*π*_(*s*_*T*_ = *s*_goal_|*s*_start_, *s*_future_) as an approximation of *p*_*π*_(*s*_*T*_ = *s*_goal_|*s*_start_, *τ*). We demonstrate the effectiveness of the proposed method through robot navigation experiments conducted in a simulated environment resembling a living room. The proposed approach can successfully navigate the robot to both near and distant states, even when conventional cost-based estimators fail.

The contributions of this study are as follows: 1) an empirical demonstration of the cost estimation model’s failure when estimating the cost between two distant states; 2) the proposal of a new evaluation metric and its estimation algorithm using deep neural network models; and 3) a demonstration of the effectiveness of our new metric through robot navigation experiments in a simulated environment resembling a real living room.

The rest of this paper is organized as follows: In Section 1, we present related works and clarify the differences between our approach and that of previous works. In Section 3, we define the proposed metric and present the cost estimation model with its training procedure for the evaluation metric. We provide our experimental results and discuss the effectiveness of our approach in Section 4. In Section 5, we draw the conclusion from our study and point out future research directions.

## 2 Related work

### 2.1 Deep image-based action generation

Because of the recent advances in deep learning, several image-based robotic action generation algorithms have been proposed. A frequently used method is an end-to-end training of a deep neural network model (Zhu et al., 2017; Codevilla et al., 2018; Kahn et al., 2018; Kumar et al., 2018; Pathak et al., 2018). These methods input images and output an action and are trained using methods such as imitation learning and reinforcement learning. However, an end-to-end model requires additional evaluations and analyses to guarantee the safety of output actions and is difficult to apply in real robotic applications. Another approach based on topological maps has also been proposed (Chaplot et al., 2020); (Shah et al., 2020). This approach constructs a topological map using images from a dataset collected in the environment and then plans actions on the topological map. However, this method also cannot guarantee the safety of planned actions because obstacle information is not reflected on the topological map.

One approach to avoiding obstacles while visually navigating a robot to its goal is visual path-following. Visual path-following tries to follow a collision-free path by tracking intermediate goal images (i.e., features or landmarks) along the path. Hirose et al. recently proposed a visual path-following algorithm that outperforms conventional visual navigation methods (Hirose et al., 2019a). A drawback of the visual path-following algorithm is that it requires collecting the image sequence of a path to follow. Another approach is to use conventional path-planning methods such as A* and rapidly exploring random tree (RRT) motion planning algorithms (LaValle, 2006) and combine conventional path planners with a CNN-based deep neural network model (Eysenbach et al., 2019; Terasawa et al., 2020; Ishihara and Takahashi, 2021). In contrast to the visual path-following method, this approach only requires a goal image and not the entire sequence of images to the goal. With the assumption that safety is critical in real-world robot applications and considering its simplicity, we focus on this approach (i.e., combining conventional path planners with CNN-based models). We will demonstrate that an evaluation metric is crucial to executing tasks when combining conventional path planners with CNN-based models. Conventionally used metrics, such as timesteps and distance between states, are not effective in navigating between distant states. Therefore, we propose a new metric and an estimation method for selecting actions. We also compare the proposed method with Hirose et al.’s visual path-following method (Hirose et al., 2019a) and demonstrate the effectiveness of our approach.

### 2.2 Evaluation metric of a robot’s action selection

Evaluation of action is the core procedure of image-based action generation and path planning. An action is generated according to the maximization or minimization of evaluation metric. Frederik et al. used pixel distance as an evaluation metric of action selection for image-based robot manipulation tasks (Ebert et al., 2017). Zhu et al. (2017) combined task-completion reward and time penalty to evaluate the action and used an image-based policy with reinforcement learning to train. Hirose et al. (2019b) combined image pixel distance, traversable probability, and velocity error to train a policy for visual path-following. However, combining multiple objectives into a single metric requires adjusting the weights to balance between the objectives. Conversely, in the context of image-based path planning, cost metrics, such as distance or time between states (Kahn et al., 2018; Eysenbach et al., 2019; Terasawa et al., 2020; Ishihara and Takahashi, 2021), are frequently used. To achieve image-based planning, the cost is frequently estimated using a neural network model (Eysenbach et al., 2019; Terasawa et al., 2020; Ishihara and Takahashi, 2021). Therefore, the accuracy of the model is critical for the success of image-based action generation. In this paper, we empirically show that when a model is trained with insufficient data, the estimation accuracy decreases proportionally to the distance between states. To overcome this issue, inspired by the work of Chebotar et al. (2021) on reinforcement learning tasks, we propose using *p*(*s*_*T*_ = *s*_goal_|*s*_start_, *τ*) the probability that the robot reaches the goal state within time *T*, as an evaluation metric of planned actions. In contrast to the results obtained by Chebotar et al. (2021), in our work, the probability is conditioned on a path *τ* instead of an action to evaluate paths.

## 3 Task achievability and its estimation method

### 3.1 Task achievability

Previous research studies focused on selecting and optimizing actions by estimating the cost to move between states, such as distance or traveling time. The cost is commonly calculated using an estimation model that consists of deep neural networks trained with data collected in the environment. However, when the training dataset contains non-optimal paths, the cost estimation becomes inaccurate, which is explained in the subsequent section. As a result of using an inaccurate cost estimator, the robot fails to achieve its tasks. To overcome this issue, we used TA as an alternative metric for selecting and evaluating actions. TA is defined as the probability that the robot will reach the goal state *s*_goal_ within time *T* with policy *π*, starting from an initial state *s*_start_ and following path *τ*:

$$p_{\pi }\left(s_{T}=s_{\text{goal}}|s_{\text{start}},\tau \right).$$ (1)

**FIGURE 2:**
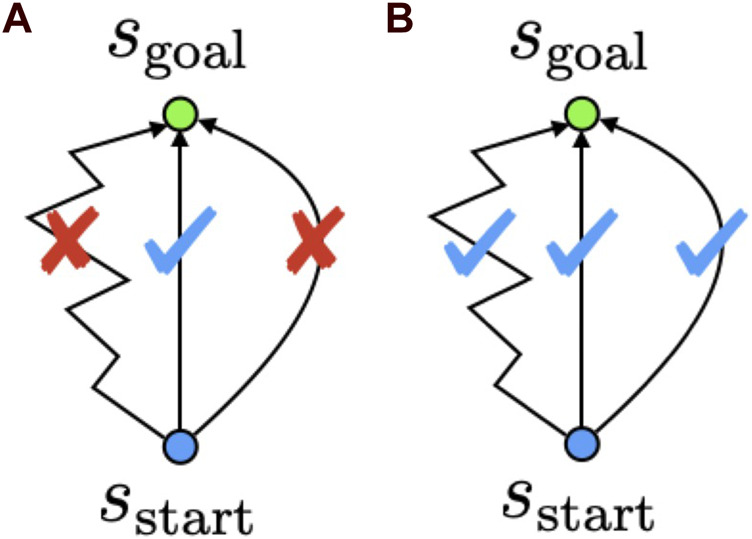
Conceptual difference between conventional cost estimation-based path planners and TA. Cost estimation-based navigation **(A)** estimates and selects the optimal cost-minimizing path. TA-based navigation **(B)** does not necessarily select the optimal cost-minimizing path and can select any path that reaches the goal.

The concept of TA is shown in Figure 2. TA-based navigation does not require estimating and selecting the optimal cost-minimizing path. Therefore, any path that reaches the goal could be selected as the candidate solution for the task. We expected this feature to improve the task execution performance of a robot. In the next subsection, we describe the proposed estimation model and training strategy used to estimate the TA.

### 3.2 Estimation model

We propose two types of TA estimation models: direct and indirect. The difference between these two models lies only in the procedure of outputting the TA. The direct model is based on a vision transformer, and it is trained to output the probability *p*_*π*_(*s*_*T*_ = *s*_goal_|*s*_start_, *τ*) directly for actions in *τ*. In contrast, the indirect estimation model consists of two components. Assuming deterministic dynamics, the indirect estimation model used a future state prediction model *s*_future_ = *f*_pred_(*τ*, *s*_start_) to predict the future state, which is then used to estimate the achievability *p*_*π*_(*s*_*T*_ = *s*_goal_|*s*_start_, *τ*) as *p*_*π*_(*s*_*T*_ = *s*_goal_|*s*_start_, *s*_future_).

#### 3.2.1 Direct estimation model

**FIGURE 3:**
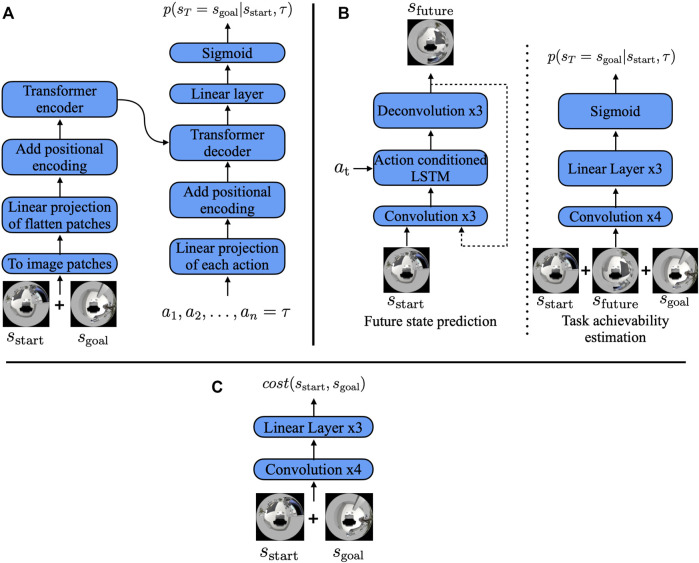
**(A)** Direct estimation model, **(B)** indirect estimation model, and **(C)** cost estimation model. The direct estimation model encoded the initial and goal state images by splitting them into image patches, as in the study by Dosovitskiy et al. (2021), and decoded them into the TA using path *τ*. The indirect estimation model first predicted the future state image and then predicted the TA with the predicted future image instead of path *τ*.

The direct estimation model is a deep neural network model based on a vision transformer (Dosovitskiy et al., 2021). Figure 3A) shows the overview of the proposed model. The *s*_start_ and *s*_goal_ images were 360° RGB images of size 120 × 120. We first concatenated these images along the color channels and split them into image patches of 12 × 12 pixels. Then, the split image patches were linearly projected onto a 128-dimensional vector and added with learnable 1D positional encoding (as in the study by Dosovitskiy et al. (2021)) before encoding was performed using a transformer. We used the two stacked transformer encoders, as presented by Dosovitskiy et al. (2021), to encode the images. Each action in path *τ* = (*a*_1_, *a*_2_, *…*, *a*_*n*_) was also linearly projected onto 128 dimensions and added with positional encoding (PE) that was generated using the sine and cosine functions from the study by Vaswani et al. (2017), which are expressed as

$$\begin{array}{cc} PE_{pos,2i} & =\sin\left(pos/10000^{2i/d_{\text{model}}}\right),\\ PE_{pos,2i+1} & =\cos\left(pos/10000^{2i/d_{\text{model}}}\right), \end{array}$$ (2)

where *pos* is the position, *i* is the dimension, and *d*_model_ is the dimension of the projected action. The output of the model was *n* number of probabilities from *p*_*π*_(*s*_*T*_ = *s*_goal_|*s*_start_, *a*_1_) to *p*_*π*_(*s*_*T*_ = *s*_goal_|*s*_start_, *a*_1_, …, *a*_*n*_). For decoding, we used two stacked transformer decoders. In addition, we used the Gaussian error linear unit (GELU) (Hendrycks and Gimpel, 2016) as the activation function for the transformer encoder and decoder. We applied the same setting used by Dosovitskiy et al. (2021) and Vaswani et al. (2017) for other parameters of transformer models.

#### 3.2.2 Indirect estimation model

Figure 3B shows the overall architecture of the indirect estimation model. The future state image prediction model consists of three blocks: the encoder, action fusion, and decoder blocks. The encoder block converts the 360° RGB images of size 120 × 120 into a feature vector $h_{t}^{\text{enc}}$ using stacked convolution layers. We used 64(6 × 6), 128(4 × 4), 128(4 × 4), and 128(4 × 4) kernels with a stride value of 2 for the convolution layers. The action fusion block combines $h_{t}^{\text{enc}}$ with the action at time *t* (i.e., *a*_*t*_) and generates a feature vector to be decoded as a *t* + 1 state image. We used action-conditioned long short-term memory (AC-LSTM) Chiappa et al. (2017) to combine the feature vector with the actions. AC-LSTM is expressed as

$$\begin{array}{cc} v_{t} & =W^{v}h_{t-1}^{\mathrm{lstm}}⊙W^{a}a_{t}\;,\\ \left[h_{t}^{\mathrm{lstm}},c_{t}\right] & =\text{LSTM}\left(h_{t}^{\mathrm{enc}},v_{t},c_{t-1}\right),\; \end{array}$$ (3)

where LSTM is the conventional LSTM layer (Hochreiter and Schmidhuber, 1997), $W^{v}\in R^{f\times f}$ , *f* denotes the number of factors (Chiappa et al., 2017), *f* = 2048, and the symbol ⊙ denotes the Hadamard product. The decoder block decodes $h_{t}^{\mathrm{lstm}}$ into the next state image *via* stepwise upsampling. The upsampling operation performs the opposite operation of the encoder block. To predict the *m* step future state, we used the predicted image as the input of the prediction model *m* times.Using the predicted future state image *s*_future_, we estimated the TA as *p*_*π*_(*s*_*T*_ = *s*_goal_|*s*_start_, *s*_future_). We concatenated the *s*_start_, *s*_future_, and *s*_goal_ state images along the color channels and input them into the stacked convolution layers, followed by the stacked liner layers. The stacked convolution layers consisted of 32(6 × 6), 32(4 × 4), 32(4 × 4), and 64(3 × 3) kernels with a stride value of 2. We used the rectified linear unit (ReLU) as the activation function for the convolution layers. The linear layers consisted of 2,048 and 1,024 hidden units with ReLU as the activation function, and the last layer output the score to feed it to the sigmoid function.

### 3.3 Model training procedure

We used a sequence of state-action tuples *D* = {(*s*_1_, *a*_1_), (*s*_2_, *a*_2_), … } collected autonomously by the robot using path planner *π* to train the future image prediction model and TA estimation model. The state was a 120 × 120 RGB image, and the action was the translational and rotational velocities of the robot: (*v*_*x*_, *v*_*y*_, *ω*). We used the modified version of RRT (RRT*) (Karaman and Frazzoli, 2011) as the path planner *π* and performed the following procedure to collect the data in the environment:

1. *m* paths are planned in the environment using *π*
2. A path is randomly selected from the *m* planned paths, and the path is followed until it ends
3. Step 1 is repeated

To plan random paths using RRT*, the tree was expanded until it reached the timeout. We did not specify the goal configuration for RRT* to generate the paths.

The training procedures we used for each model presented in the previous subsections are presented in the subsequent sections.

#### 3.3.1 Estimation model training procedure

Because *p*_*π*_(*s*_*T*_ = *s*_goal_|*s*_start_, *τ*) is a binary classifier, we performed maximum likelihood estimation for the model parameters. The training data were labeled as follows:

1. A state is sampled from training data *D*, and the sampled state is used as *s*_start_.
2. A step number *t* (1 ≤ *t* ≤ *T*) is sampled, and the state *t* steps ahead from *s*_start_ is used as *s*_goal_.
3. (Direct estimation model) All the actions from 1 to *t* are labeled as positive, and all the actions from *t* + 1 to *n* are labeled as negative, where *n* denotes the number of actions required to input into the transformer model. (Indirect estimation model) A step number *t*′ (1 ≤ *t*′ ≤ *T*) is sampled, and the state *t*′ steps ahead from *s*_start_ is used as *s*_future_. The action is labeled as positive if *t*′ is between 1 and *t* and labeled as negative otherwise.

In addition to the aforementioned labeling, we augmented the training batch by swapping *s*_start_ and *s*_goal_ and labeling them as negative. We used cross-entropy loss as the loss function. Adam (Kingma and Ba, 2015) was used as the optimizer, and learning rates of 2.0 × 10^−3^ and 1.0 × 10^−4^ were used for the direct and indirect estimation models, respectively. For both models, the batch size was set to 32, *n* was set to 100, and *T* was set to 1,500.

#### 3.3.2 Image prediction model training procedure

We trained the image prediction network by minimizing the sum of the mean squared error *via* *K*-step prediction (Oh et al., 2015). The latter is expressed as

$$\begin{array}{c} \frac{1}{2K}\underset{t}{\sum }\overset{K}{\underset{k=1}{\sum }}{\left\|s_{t+k}^{\left(i\right)}-f_{\text{pred}}\left({\hat{s}}_{t+k-1}^{\left(i\right)},a_{t+k-1}^{\left(i\right)}\right)\right\|}^{2}, \end{array}$$ (4)

where ${\hat{s}}_{t+k}^{\left(i\right)}$ denotes the *k*-step prediction for the *i*th training data starting from time *t*. The loss was propagated for 150,000 iterations per prediction step *K*, which was increased stepwise, starting from 1 up to 9 in increments of 2 (i.e., 1, 3, 5, 7, and 9 steps). We used the Adam optimizer (Kingma and Ba, 2015) with a learning rate of 1.0 × 10^−4^ for *K* = 1 and 1.0 × 10^−5^ for the other values of *K*. The batch size was set to 4. Furthermore, as in the study by Oh et al. (2015), we unrolled the network through 10 steps and propagated the error for 10 predicted frames when the prediction step was *K* = 1, in which the reference image was provided as an input instead of the network output.

## 4 Experiments

### 4.1 Empirical evaluation of the cost estimation model

#### 4.1.1 Cost estimation model

For the experiment, we trained the cost estimation model shown in Figure 3C. The model’s architecture is identical to the indirect estimation model’s architecture. The model’s convolution layer consists of 32(6 × 6), 32(4 × 4), 32(4 × 4), and 64(3 × 3) kernels with a stride value of 2. The linear layers consist of 2,048 and 1,024 hidden units, and it output the cost, which is the estimated number of timesteps required to move from *s*_start_ to *s*_goal_. We used ReLU as the activation function for each layer except for the last linear layer, and we trained this network using the dataset mentioned in the previous section. We focused on the steps between the two states in the dataset to train the cost estimation network without explicitly providing the cost as a label. Specifically, we randomly sampled two states in the dataset, $s_{t_{1}}$ and $s_{t_{2}}\;\left(0\leq t_{1}\leq t_{2}\leq T\right)$ , and computed the difference between their timesteps as Δ*t* = *t*_2_ − *t*_1_. This Δ*t* was the cost to move between states $s_{t_{1}}$ and $s_{t_{2}}$ . Because the data were collected along the path generated by the asymptotically optimal path planner RRT*, we assumed that this timestep difference was almost the optimal cost-minimizing path between the states. We trained the network by minimizing the loss function that represents the mean squared error computed using *N* randomly sampled pairs of states in the dataset. This loss function is expressed as

$$\begin{array}{c} \frac{1}{N}\overset{N}{\underset{n=1}{\sum }}{\left\|{\Delta t}^{\left(n\right)}-f_{\text{cost}}\left(s_{t_{1}}^{\left(n\right)},s_{t_{2}}^{\left(n\right)}\right)\right\|}^{2}, \end{array}$$ (5)

where *f*_cost_ is the modeled cost function, Δ*t* ^(*n*)^ is the distance in steps, and $s_{t_{1}}^{\left(n\right)}$ and $s_{t_{2}}^{\left(n\right)}$ are the two states in the *n*th sample. We trained the network over 1 M iterations using the Adam optimizer with a learning rate of 1.0 × 10^−3^ for the first 0.1 M iterations and a learning rate of 1.0 × 10^−4^ for the remaining iterations. The batch size *N* was set to 32.

#### 4.1.2 Evaluation results of the trained model

**FIGURE 4:**
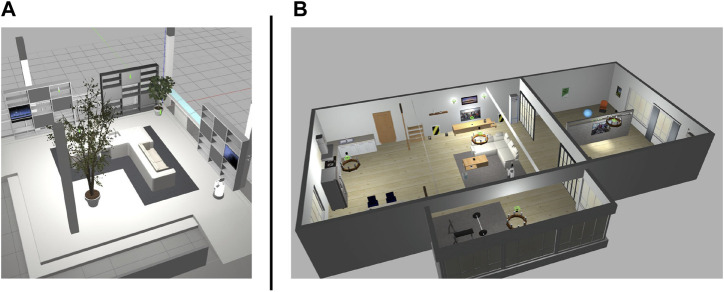
Simulation environments. Megaweb (2015) **(A)** and AWS small house **(B)**.

**FIGURE 5:**
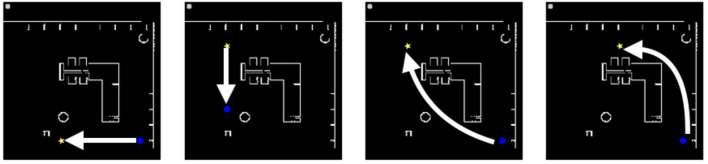
Reference paths 1 to 4 from the left (the shortest paths between the initial and goal states) in the “Megaweb 2015” environment. The blue circle and yellow star denote the initial and goal positions, respectively.

**FIGURE 6:**
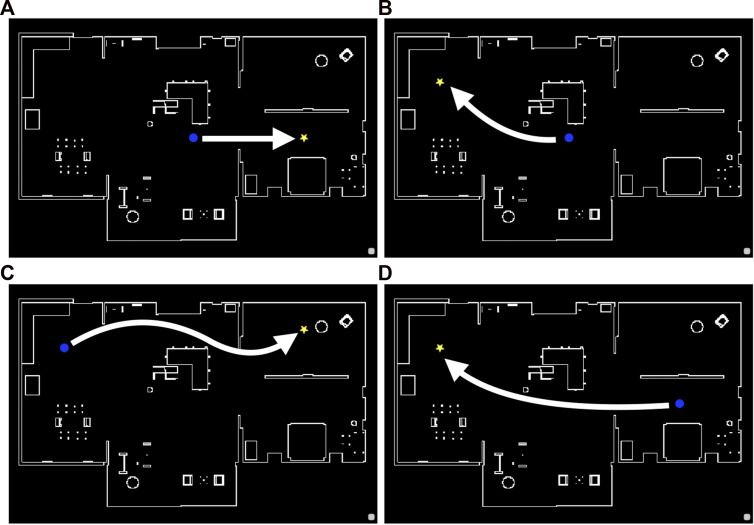
Reference paths 1 **(A)**, 2 **(B)**, 3 **(C)**, and 4 **(D)** (the shortest paths between the initial and goal states) in the “AWS small house” environment. The blue circle and yellow star denote the initial and goal positions, respectively.

**FIGURE 7:**
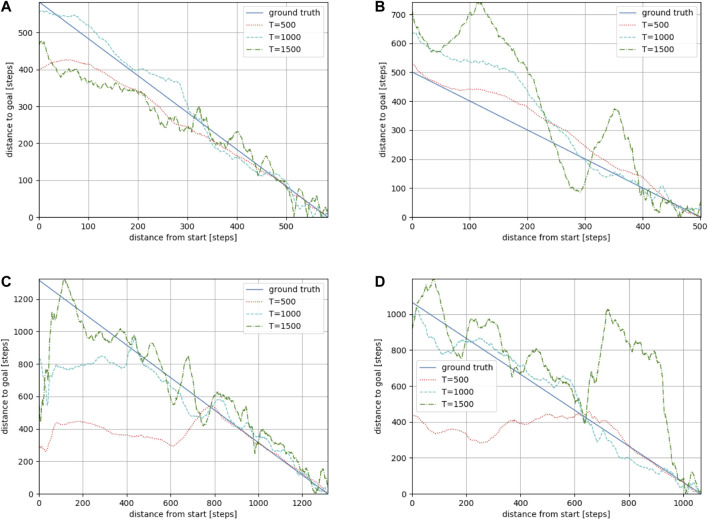
Estimation results along reference paths 1 **(A)**, 2 **(B)**, 3 **(C)**, and 4 **(D)** in the “Megaweb 2015” environment. The larger the *T*, the longer the distance that could be predicted. However, a large *T* resulted in an unstable estimation.

**FIGURE 8:**
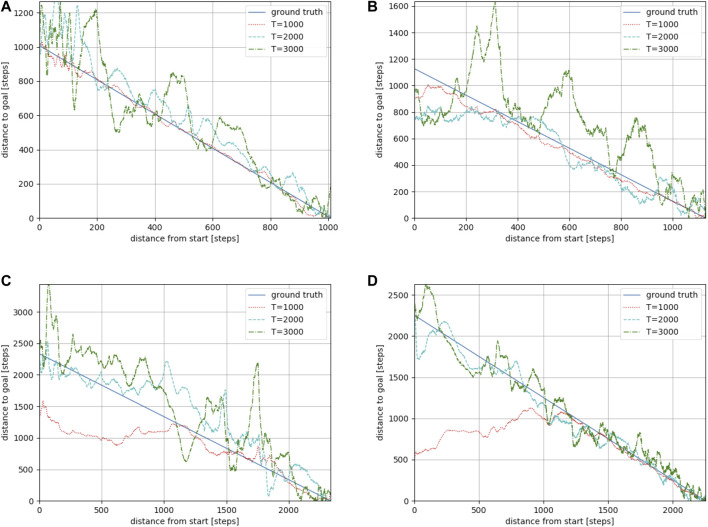
Estimation results along reference paths 1 **(A)**, 2 **(B)**, 3 **(C)**, and 4 **(D)** in the “AWS small house” environment. The larger the *T*, the longer the distance that could be predicted. However, a large *T* resulted in an unstable estimation.

We used Toyota’s Human Support Robot (HSR), a common robotics platform developed for researchers (Yamamoto et al., 2018), simulated in Gazebo (Koenig and Howard, 2004), an open-source 3D dynamic multi-robot environment simulator. We also used the “Megaweb 2015” (Toyota Motor Corporation, 2015) and “AWS small house” (AWS RoboMaker, 2022) world as the simulation environment. Both are publicly available environments and are not specially designed for our experiment. Figure 4 presents an overview of both environments. The “Megaweb 2015” environment’s size is 7.0 m × 7.0 m, and it contains 12 objects. The dimension for the “AWS small house” is 20.0 m × 10.0 m, and 87 objects are available in the environment. We collected the training data for approximately 2 h in each environment and trained the cost estimation model for each environment with three different values of *T* (defined as the maximum number of steps between sampled states during the training): 500, 1,000, and 1,500 for “Megaweb 2015” and 1,000, 2,000, and 3,000 for “AWS small house.” Therefore, the larger the *T*, the longer the distance that could be predicted. To evaluate the cost estimation model, we defined reference paths shown in Figures 5, 6 and compared the estimation quality of the estimation model to the ground truth value.Each reference path was generated using the RRT* path planner. The estimation results of the trained models are shown in Figures 7, 8. The figure demonstrates that, in both environments, for the model trained with a small *T* value, the estimation failed for steps larger than *T* but was stable for steps shorter than *T*. In contrast, the estimation model trained with a large *T* value was unstable for all the steps. This was because for larger *T* values, more incorrectly labeled training data could be generated (Figure 1). To correctly train the model, collecting a large amount of training data and accurately labeling data during training is necessary. However, we assumed that meeting such requirements would be unrealistic in real-world robotic applications.

### 4.2 Navigation experiment

To demonstrate the effectiveness of the proposed metric, we conducted a navigation experiment using the navigation algorithm shown in Algorithm 1.

Algorithm 1 Navigation algorithm.

1: Observe initial state *s* and set the goal state to *s*_goal_
2: **while** *ϵ* < *f*_cost_(*s*, *s*_goal_) **do**
3: Sample *M* collision-free paths *τ*_1_, …, *τ*_*M*_ with the planner *π*
4: **for** *i* = 1 **to** *i* ≤ *M* **do**
5:  Compute the input sequence *u*_1:*T*,*i*_ to follow *τ*_*i*_
6:  Compute cost $c_{\tau_{i}}=f_{\text{cost}}\left(s_{\text{goal}},f_{\text{pred}}\left(\tau_{i},s_{\text{start}}\right)\right)$ or TA $p_{\tau_{i}}=p_{\pi }\left(s_{T}=s_{\text{goal}}|s_{\text{start}},\tau_{i}\right)$

7: **end for**
8: **if** Cost is computed **then**
9: $\tau^{*}=\arg\min_{\tau_{i}}c_{\tau_{i}}$

10: **else**
11: $\tau^{*}=\arg\max_{\tau_{i}}p_{\tau_{i}}$

12: **end if**
13: Follow τ* and observe the next state *s*_next_
14: *s* ← *s*_next_
15: **end while**

The navigation algorithm generated multiple paths using the RRT* path planner (Karaman and Frazzoli, 2011) and selected a path that either minimizes or maximizes the predicted metric (i.e., cost or TA). We set the number of samples *M* in the navigation algorithm to 32 in the experiment. The experiment was conducted in the same environment and with the same HSR robot demonstrated in Figure 4. We used the same dataset mentioned in the previous section to train the models. The robot’s maximum translational and rotational speed was set to 0.2 m/s and 0.5 rad/s, respectively. Following the experiment described in the previous section, we defined four combinations of initial and goal positions and evaluated the navigation performance of the robot. In addition, considering the stochasticity of the RRT* path planner, we conducted 10 navigation trials for each combination of initial and goal positions. We chose two navigation methods as baseline algorithms. One algorithm is the deep visual model predictive control (DVMPC) (Hirose et al., 2019a) algorithm, one of the state-of-the-art visual path-following algorithms. The other is Algorithm 1, executed using a conventional cost estimator. As in our algorithm, DVMPC uses 360° images as input. However, DVMPC uses the Ricoh THETA S camera (Ricoh Company, Ltd, 2022) to capture 360° camera angles. Therefore, we simulated the Ricoh THETA S camera by placing two 360° cameras on the front and back sides of the robot.

**FIGURE 9:**
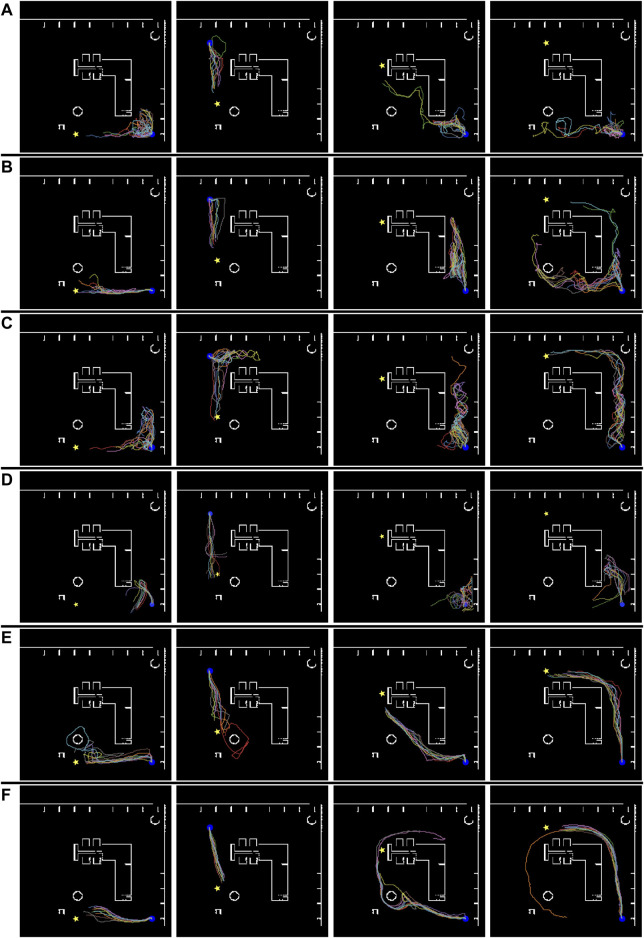
Simulated robot trajectories in the “Megaweb 2015” environment. **(A)** Conventional cost estimator trained using *T* = 500. **(B)** Conventional cost estimator trained using *T* = 1,000. **(C)** Conventional cost estimator trained using *T* = 1,500. **(D)** DVMPC. **(E)** Proposed task achievability (indirect estimation model). **(F)** Proposed task achievability (direct estimation model). The blue circle and yellow star denote the initial and goal positions, respectively.

**FIGURE 10:**
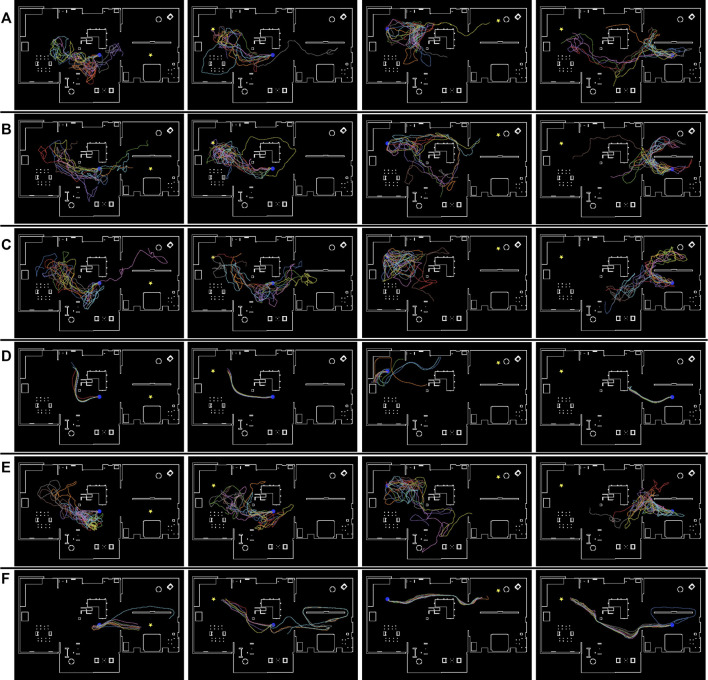
Simulated robot trajectories in the “AWS small house” environment. **(A)** Conventional cost estimator trained using *T* =500. **(B)** Conventional cost estimator trained using *T* = 1,000. **(C)** Conventional cost estimator trained using *T* = 1,500. **(D)** DVMPC. **(E)** Proposed task achievability (indirect estimation model). **(F)** Proposed task achievability (direct estimation model). The blue circle and yellow star denote the initial and goal positions, respectively.

Figure 9 illustrates the navigation trajectory of the robot in the “Megaweb 2015” environment. Figure 9 demonstrates that the navigation based on the cost estimation succeeded only when the distance between the initial and goal positions was short. DVMPC succeeded only with paths that did not require large changes in its orientation. In contrast, Figure 9 shows that the navigation based on TA succeeded in all the navigation scenarios. In addition, the navigation trajectory computed using the direct estimation model was smoother compared to that of the indirect estimation model. This was because the direct estimation model output the probability with a single forward computation of the network, and the robot was controlled with a shorter interval. Furthermore, even though the proposed metric did not guarantee the optimality of the path, the path generated by TA was identical to the shortest route to the goal (Figure 5). There are two possible reasons for this result. First, the navigation algorithm selected a path using TA, which was generated by the optimal path planner RRT*. Second, high TA states were concentrated around optimal cost-minimizing paths because the training data were sampled using RRT*. Figure 10 illustrates the navigation trajectory of the robot in the “AWS small house” environment. From Figure 10, because the “AWS small house” environment is larger than the “Megaweb 2015” environment, we can find that navigation based on the cost estimation failed in most of the tasks. In addition, as in “Megaweb 2015”, DVMPC has succeeded only with paths that do not require large changes in orientation. We found that once DVMPC diverges from a reference path, it cannot recover from divergence and fails following the path. Please check the Supplementary Appendix for further comparison results between DVMPC and our proposed algorithm. In contrast, the navigation based on TA with the indirect model did not succeed navigating, but navigation based on TA with the direct model succeeded in all the navigation scenarios. We confirmed that navigation based on TA with a direct model has a success rate of 70%, even though the goal position was selected at random in the “AWS small house” environment. Additional experimental results can also be found in Supplementary Appendix. These experimental results verify the effectiveness of the proposed approach and the instability of cost estimation-based navigation.

## 5 Conclusion and future research directions

In this research, due to the strong demand for image-based technologies that can easily enable multiple robotic agents to be managed and controlled visually, we studied an evaluation metric for the action selection of image-based robot navigation algorithms. In previous research, the optimal cost to move between two states, such as the shortest distance or time, was used to select robot actions in image-based path-planning algorithms. To estimate the optimal cost-minimizing path using images, a deep neural network-based model is widely used. In this study, we empirically demonstrated that when an estimator is trained with randomly collected trajectory data, the accuracy of the optimal cost-minimizing path estimation depends on the maximum predicted distance. In general, navigation algorithms based on inaccurate estimators fail to navigate a robot when the goal state is at a large distance from the initial state. To overcome this issue, we proposed task achievability as an alternative cost metric for evaluating robot actions in image-based path-planning algorithms. To estimate the TA, we proposed a direct approach and an indirect approach. The direct approach estimated the TA by training a vision transformer-based model, which output the metric directly from the input images. The indirect approach first predicted a future state image according to the planned path and then used the predicted future state image to estimate the TA. Through navigation experiments conducted in a simulated environment resembling a living room, we demonstrated that path planning using our new metric succeeded in navigating the robot, even when conventional cost estimation-based and visual path-following approaches failed. Especially, the direct approach robustly worked among different environments and achieved a 70% navigation success rate on average.

However, in our experiment, the navigation of the robot was not smooth, and oscillations were observed. We will work on this issue in our future work. Possible approaches could be as follows:

• Applying filtering methods such as the Kalman filter to smooth the velocity input
• Generating navigation paths taking into consideration both the position and velocity of the robot
• Improving the estimator’s accuracy to avoid selecting incorrect navigation paths

This will be one of the directions to take the study forward. Improving the estimator’s robustness and accuracy against real images, lighting conditions, and new environments will also be a future research direction. Furthermore, applying the proposed metric to other robotic actions, such as manipulation tasks, and showing the effectiveness of the approach with such tasks is also an interesting future research work direction.

## Data Availability

The raw data supporting the conclusion of this article will be made available by the authors, without undue reservation.
