# Supplementary material for: Image-based robot navigation with task achievability
Source: Front Robot AI. 2023 May 31;10:944375. doi: 10.3389/frobt.2023.944375 (PMC10264687; doi:10.3389/frobt.2023.944375)
Supplement: Supplementary file 1 [file Presentation1.pdf]

Image-based Robot Navigation with Task Achievability (Appendix)

Yu Ishihara [1,*] **and Masaki Takahashi**[2]

[1] *Graduate School of Science and Technology, Keio University, 3-14-1 Hiyoshi, Kohoku-ku, Yokohama 223-8522, Japan*
[2] *Department of System Design Engineering, Keio University, 3-14-1 Hiyoshi, Kohoku-ku, Yokohama 223-8522, Japan*

Correspondence*:
Yu Ishihara
yu_ishihara.a2@keio.jp

APPENDIX

A.1 Performance Comparison Result against DVMPC

We conducted two experiments, straight navigation and initial orientation, to further compare the navigation performance between our proposed method (direct TA estimation) and DVMPC. We used "AWS small house" environment in the experiments. Straight navigation experiment evaluates the navigation performance on a straight line to the goal and initial orientation experiment evaluates the effect, on navigation performance, of initial orientation of the robot. See Fig. A.1 for the overview of navigation tasks conducted in each experiment.

Straight Navigation Experiment

The aim of this experiment is to evaluate the pure navigation performance of each algorithm when no obstacles are on the path. We designed this experiment because DVMPC failed navigating reference path 3 in Fig. 6, which is smooth, and no large orientation changes were necessary. Fig. A.2 shows the navigation trajectories of the robot during the experiment. We conducted 10 trials for each navigation. From the figure, we can confirm that both methods achieve arrival to the goal in this simple experiment. DVMPC can arrive to the goal without changing the robot's direction because DVMPC tries to follow a

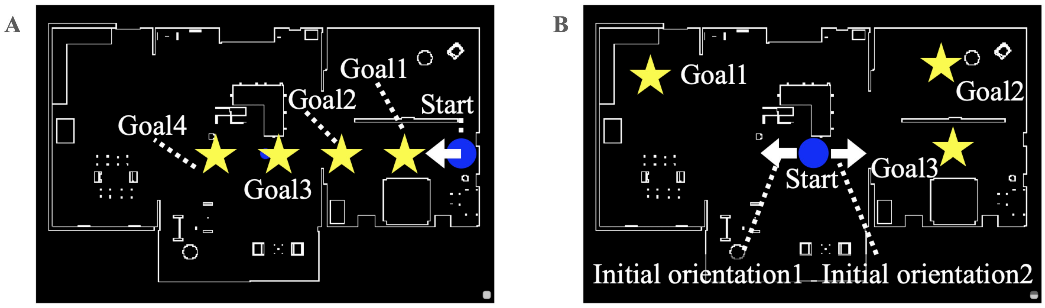

Figure A.1. Overview of (**A**) straight navigation experiment and (**B**) initial orientation experiment. (**A**) Robot starts navigating from the right edge of the environment to each goal. There are no obstacles between the start and goal positions. (**B**) Robot starts navigating from the center of the environment to each goal with two different initial orientations.

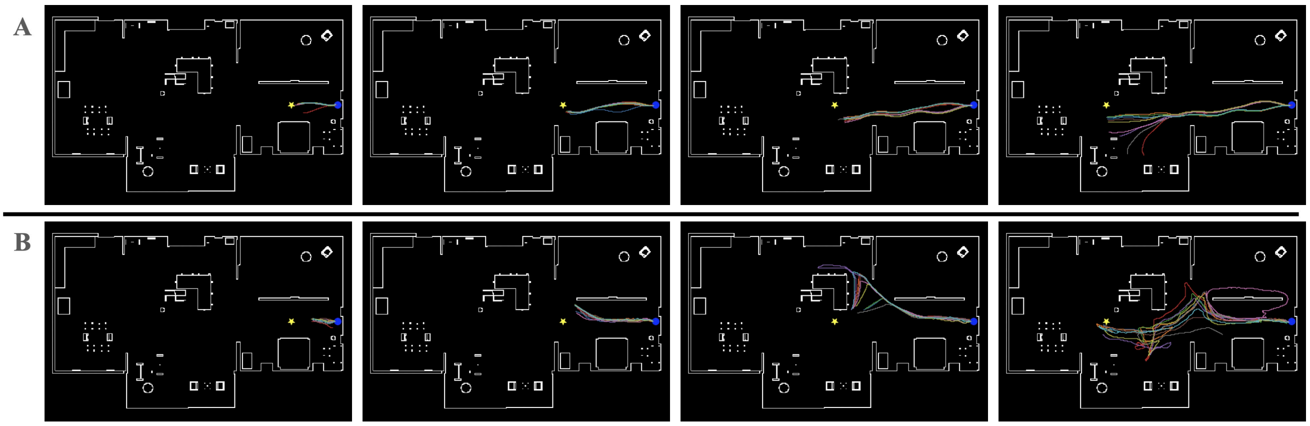

Figure A.2. Navigation trajectory in straight navigation experiment (**A**) DVMPC and (**B**) Proposed method. The blue circle and yellow star respectively denote the initial and goal positions. The whiteW arrow denotes the initial orientation of the robot.

Table A.I. Quantitative results of each navigation task in straight navigation experiment.

Task	Method	Success rate	Distance from goal [m]	Time [s]
To goal 1 (orientation 1)	DVMPC	100%	0.5 ± 0.2	12 ± 0
	TA (direct)	100%	1.4 ± 0.1	12 ± 1
To goal 1 (orientation 2)	DVMPC	100%	0.5 ± 0.1	29 ± 0
	TA (direct)	100 %	1.3 ± 0.1	45 ± 3
To goal 2 (orientation 1)	DVMPC	100%	1.1 ± 0.1	43 ± 0
	TA (direct)	80 %	1.5 ± 0.1	75 ± 17
To goal 2 (orientation 2)	DVMPC	70%	1.0 ± 0.3	61 ± 0
	TA (direct)	60 %	0.6 ± 0.3	149 ± 28

trajectory of subgoal images. In contrast, the proposed method sometimes failed reaching the goal directly from the start position. However, the proposed method can recover from an incorrect position and reach the goal. Table A.I shows the quanititative results of the experiment. We counted the navigation as success when the final position of the robot was within 2 m from the goal position. We set the threshold to 2 m because the goal image taken 1 m away from the goal was almost identical to the goal image. From the Table, we can also confirm that both methods succeed in navigating to the goal in these simple tasks.

Initial Orientation Experiment

Fig. A.3 shows the navigation result of proposed method and DVMPC in the initial orientation experiment. Similar to the straight navigation experiment, we conducted 10 trials for each navigation. From the figures, we can confirm that DVMPC can only navigate to the goal when the initial orientation of the robot is directed towards the goal. This is because, DVMPC cannot make a pivot and turn without the robot diverging from its reference trajectory. In contrast, with our proposed method, the robot succeeds in reaching the goal in both initial orientations. Our proposed method achieves reaching the goal even in the case where the robot moves away from the goal at the beginning of the navigation. Table A.II shows the quantitative results of the experiment. From the table, we can also confirm that our proposed method is sufficiently robust to accommodate any initial orientation of the robot. Our proposed method achieves reaching the goal position in all the navigation tasks in constrast to DVMPC, which only achieved reaching the goal in the half of navigation tasks.

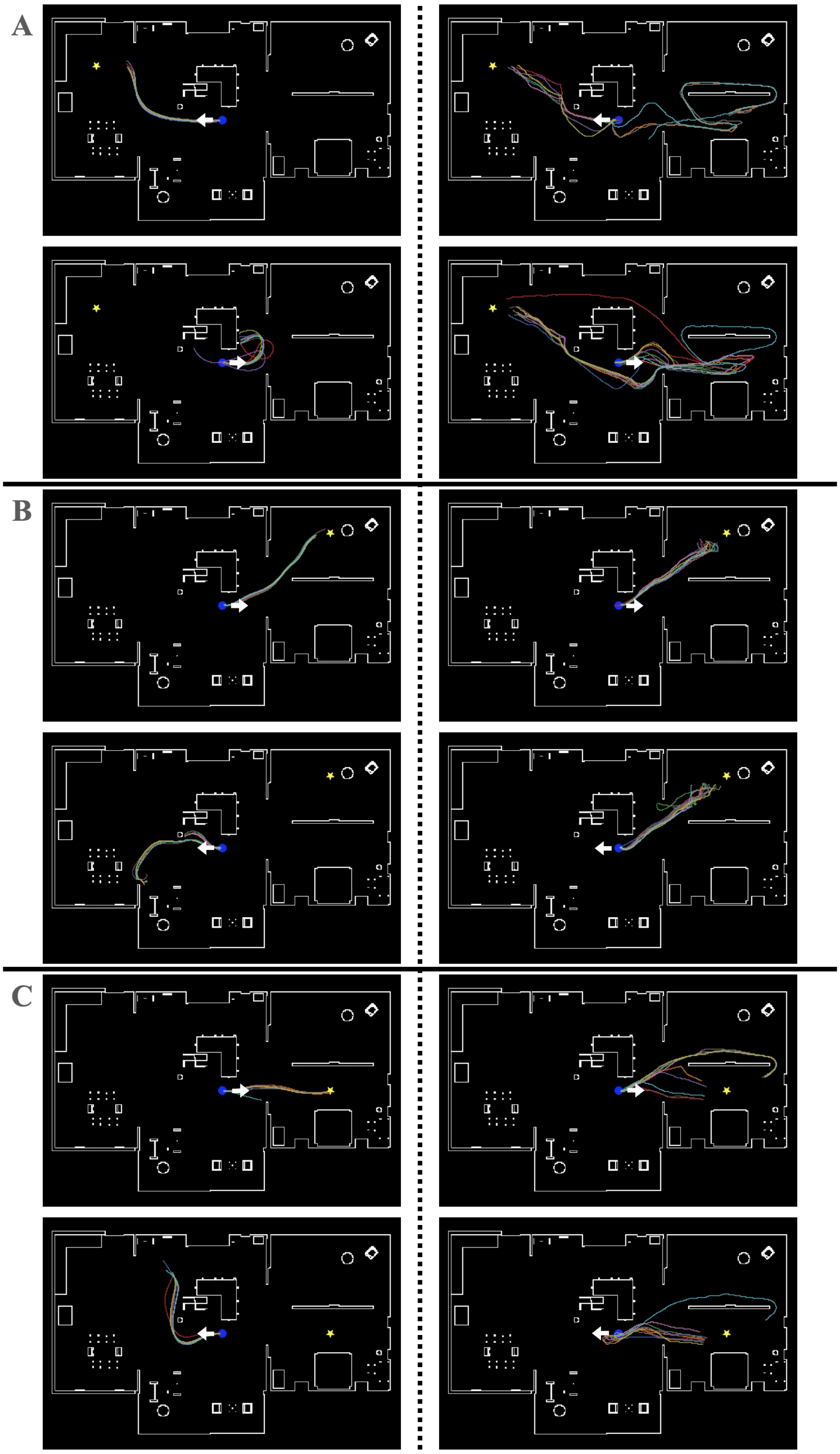

Figure A.3. Navigation trajectories of DVMPC (Left) and proposed method (Right) in initial orientation experiment. (**A**) Trajectory of each method to goal 1. (**B**) Trajectory of each method to goal 2. (**C**) Trajectory of each method to goal 3. The blue circle and yellow star respectively denote the initial and goal positions. White arrow denote the initial orientation of the robot.

Table A.II. Quantitative results of each navigation task in initial orientation experiment.

Task	Method	Success rate	Distance from goal [m]	Time [s]
To goal 1 (orientation 1)	DVMPC	80%	1.8 ± 0.1	34 ± 0
	TA (direct)	70 %	1.3 ± 0.2	49 ± 4
To goal 1 (orientation 2)	DVMPC	0%	-	-
	TA (direct)	90 %	1.3 ± 0.2	126 ± 33
To goal 2 (orientation 1)	DVMPC	0%	-	-
	TA (direct)	100 %	1.2 ± 0.4	66 ± 23
To goal 2 (orientation 2)	DVMPC	100%	0.8 ± 0.2	33 ± 0
	TA (direct)	100 %	1.1 ± 0.3	54 ± 14
To goal 3 (orientation 1)	DVMPC	0%	-	-
	TA (direct)	90 %	1.3 ± 0.1	42 ± 5
To goal 3 (orientation 2)	DVMPC	90%	0.2 ± 0.0	29 ± 0
	TA (direct)	50 %	1.3 ± 0.7	31 ± 3

B.2 Experimental Details of Random Goal Position Experiment

In this section, we describe the details of the random goal position experiment mentioned in the Experiments section. To verify the average navigation performance of our proposed method (direct TA estimation), we measured the success rate of navigation from a start position to a randomly selected goal position. We first placed the robot in one of four start positions, shown in Fig. 6, and conducted a navigation to a randomly selected goal position. To select a feasible goal position, we first generated a path from a start position using the RRT* path planner algorithm, and used the final position of the path as the goal position. To sample a random path using RRT*, the tree was expanded until it timed out. We did not specify the goal configuration for RRT* to generate a random path. Timeout was set to 200 ms. For each starting position, we selected 100 random goal positions and measured the navigation success rate. We also selected an initial orientation for the robot by uniformly sampling orientations θ between $0.0 \leq \theta \leq 360.0$ degrees. We considered the navigation successful when the final position of the robot was within 2 m from the goal position, as was done in the previous experiments.

The success rate of navigation to randomly selected goal positions was 58% for position 1, 74% for position 2, 71% for position 3, and 75% for position 4. We can confirm that the average success rate of navigation with our proposed method is around 70%. However, we found that 15% of the navigation failed because of the cost estimation in line 2 of Algorithm 1. The robot failed to stop around the goal and continued to find the goal. Therefore, we assume that potential success rate of our proposed method is around 85%.
